# Derivation of a Novel CIHI in Patients with Lung Adenocarcinoma for Estimating Tumor Microenvironment and Clinical Prognosis

**DOI:** 10.1155/2021/4495489

**Published:** 2021-11-22

**Authors:** Liang Zhou, Guangyan Xu, Li Wang, Jianyong Zhang, Weimin Li

**Affiliations:** ^1^Department of Respiratory and Critical Care Medicine, West China Hospital of Sichuan University, Chengdu, Sichuan 610041, China; ^2^Department of Respiratory and Critical Care Medicine, Affiliated Hospital of Zunyi Medical University, Zunyi, Guizhou 563000, China

## Abstract

An interaction between hypoxia and immunity has been confirmed in tumor tissue. However, there is no combined biomarker for diagnosis on this basis. Therefore, we developed a scoring formula based on markers of hypoxia and immunity. Firstly, the hypoxia-immune formula of lung adenocarcinoma (LUAD) was derived using LASSO-Cox regression in three cohorts from public database, and the corresponding score was calculated for each patient. The formula is as follows: combined hypoxia and immune index (CIHI) = LDHA expression × 0.2252 + GAPDH expression × 0.0727 + ANGPTL4 expression × 0.0724 + VEGFC expression × 0.1911 + DKK1 expression × 0.1355 + ADM expression × 0.0588 + BTK expression × −0.1659. Meanwhile, patients were divided into groups according to high and low CIHI, and expression profiles of hypoxia markers and immune markers were analyzed in different groups. CIHI was used to confirm that patients with high CIHI represented a state of hypoxia^high^-immunity^low^, which had worse overall survival. We also discussed the evaluation value in the immune microenvironment and clinical application of CIHI. In conclusion, this study developed and validated a hypoxia-immune formula that can guide hypoxia modifier treatment and immunotherapy in LUAD.

## 1. Introduction

There are many different types of cancer, but lung cancer is the most common cause of cancer-related death worldwide [1]. Non-small-cell lung cancer accounts for four-fifths of all lung cancer cases, with lung adenocarcinoma (LUAD) the most frequent subtype [2]. Unfortunately, early-stage patients with LUAD are difficult to diagnose, and the condition is frequently advanced by the time they are diagnosed [3].

The tumor microenvironment is closely related to the degree of tumor development. A rising number of studies have discovered that anomalies in tumor metabolism are linked to alterations in the tumor microenvironment. Immunotherapy refers to the targeting of immune checkpoint, such as PD-1/PD-L1 and CTLA-4; the infiltration of immune cells is a critical component determining the efficacy of this treatment [4]. In addition, immunosuppressive metabolites can be produced in a process that inhibits their antitumor activity, while immune escape can be facilitated by affecting the expression of cell surface markers as a method. And immune checkpoint blockade [5], such as B7-H3 and PD-1 [6], can help restore glucose in the tumor microenvironment (TME), allowing cytokine production and glycolysis [7]. Endogenous tumor metabolism can be targeted to boost immune responses [8]. In combination with immune checkpoint inhibitors, targeted metabolism is extremely likely to be a new immunotherapy strategy to overcome immune resistance [9]. Hypoxia-related mechanisms have long been one of the hallmarks of cancer signaling pathways. Hypoxia is a common occurrence in solid tumors, and it has been linked to cancer metastasis, extracellular matrix structure, angiogenesis, stem cell characterization, and metabolic reprogramming [10]. A lot of previous research has looked into the link between the TME and hypoxia [11]. The hypoxic response of T cells, for example, aids immunotherapy by increasing CD137 expression [12]. Another example is glycolysis in the presence of sufficient oxygen in breast cancer cells, which can be regulated by macrophage-related lncRNAs [13]. Furthermore, inhibiting NRF1 degradation under hypoxic settings has a negative impact on tumor-associated macrophage polarization [14].

As a result, we aimed to develop and validate an integrated index of immunity and hypoxia in this study in order to estimate the microenvironment of LUAD. We also discussed the evaluation value in the immune microenvironment and clinical application value of CIHI.

## 2. Materials and Methods

### 2.1. LUAD Patient Datasets and Hypoxia-Immune Genes

We download clinical data and RNA-seq for LUAD cases in TCGA and GEO database. Excluding missing data, 529 patients from TCGA-LUAD project, 398 patients from GSE68465 dataset, and 442 patients from GSE72094 dataset were finally included in this study. Annotate gene names with their respective platform files in three cohorts. Meanwhile, we searched 200 hypoxia-related genes [15] and 2483 immune-related genes [16] in the previous references. Considering the different gene annotation of three datasets, we finally identified 1220 immune-hypoxia-related genes.

### 2.2. Calculating CIHI

In the study, we classified all LUAD cases into three sets, including test set (GSE68465 and GSE72094) and training set (TCGA) to improve the confidence of CIHI. We used both sets to validate the prediction performance, while one of the training sets was used to construct the prognostic prediction model. Firstly, based on the 1220 immune-hypoxia-related genes in the training set, we identified significant prognostic genes by univariate Cox regression analysis. Subsequently, we used the glmnet package to perform LASSO regression and Cox regression for screening genes participating in the CIHI formula. Meanwhile, we used multivariate Cox regression analysis in order to construct a formula for generating coefficient of each gene. Here is the formula for calculating CIHI: (gene 1 expression × coefficient) + (gene 2 expression × coefficient) + ⋯+(gene *n* expression × coefficient). Also, all cases were divided into two groups (low-CIHI group or high-CIHI group) according to the median of the CIHI scores. In addition, our signatures were validated using the training set and the test set described above.

### 2.3. Clinical Benefit Assessment

The training set and validating set were divided into the high-CIHI and low-CIHI groups according to the median value of the CIHI score. The Kaplan-Meier curves and ROC analysis were used to predict OS of three cohorts for LUAD patients (1-year OS, 3-year OS, and 5-year OS, respectively). We then used calibration curve of survival to validate the accuracy of CIHI. In addition, we plotted heatmaps of clinicopathological factors with CIHI scores to calculate their correlations.

### 2.4. The Stromal and Immune Infiltration in the Tumor Environment

The tumor microenvironment contains various stromal cells and immunocytes. We estimated the stromal infiltrating via calculation of stromal score and tumor purity by Estimation of STromal and Immune cells in MAlignant Tumor tissues using Expression data (ESTIMATE) [17]. Besides, the infiltration level differences between the high-CIHI and low-CIHI groups were compared using the myeloid lineage phenotypic and functional markers, inhibitory immune receptors or ligand markers, activating immune receptor markers, IFN*γ* signature markers, and immune modulator markers.

### 2.5. Statistical Analyses

The statistical analyses were conducted in the R software (version 4.0.1). A two-sided *p* value < 0.05 was regarded as statistically significant. The log-rank test was used for the Kaplan-Meier curves of TCGA and GEO dataset patient survival analyses. For normally distributed variables, we used Student's *t*-test to conduct the pairwise comparisons, and for nonnormally distributed variables, the Wilcoxon test was performed. Finally, the Spearman correlation analysis was used to compute the significance of correlations between CIHI and genes. ∗∗∗, ∗∗, ∗, and NS refer to *p* < 0.001, <0.01, <0.05, and not significant, respectively.

## 3. Results

### 3.1. A Landscape of Immune and Hypoxia Gene Profiles in Different Cohorts

**Figure 1:**
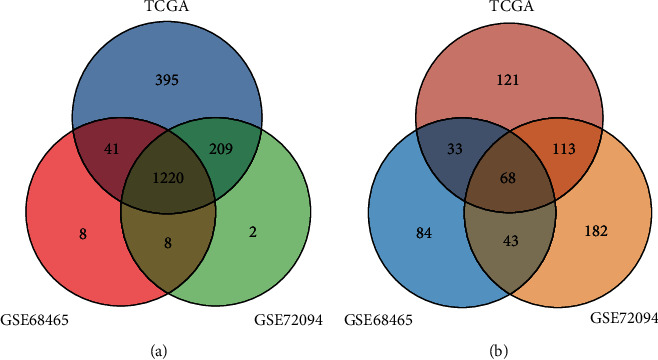
Landscape of immune and hypoxia gene profiles. (a) 1220 immune-hypoxia-related genes annotated in three cohorts. (b) Prognostic genes in each cohort were calculated, and 68 common prognostic genes were identified.

Excluding missing data, 529 patients from TCGA-LUAD project, 398 patients from GSE68465 dataset, and 442 patients from GSE72094 dataset were finally included in this study. Considering the different gene annotation of three datasets, we finally identified 1220 immune-hypoxia-related genes (Figure 1(a)). Subsequently, univariate Cox regression analysis was used to reveal the prognostic genes, and 68 genes were identified in TCGA, GSE68465, and GSE72094 cohorts (Figure 1(b)).

### 3.2. CIHI Was Calculated for Each Patient

**Figure 2:**
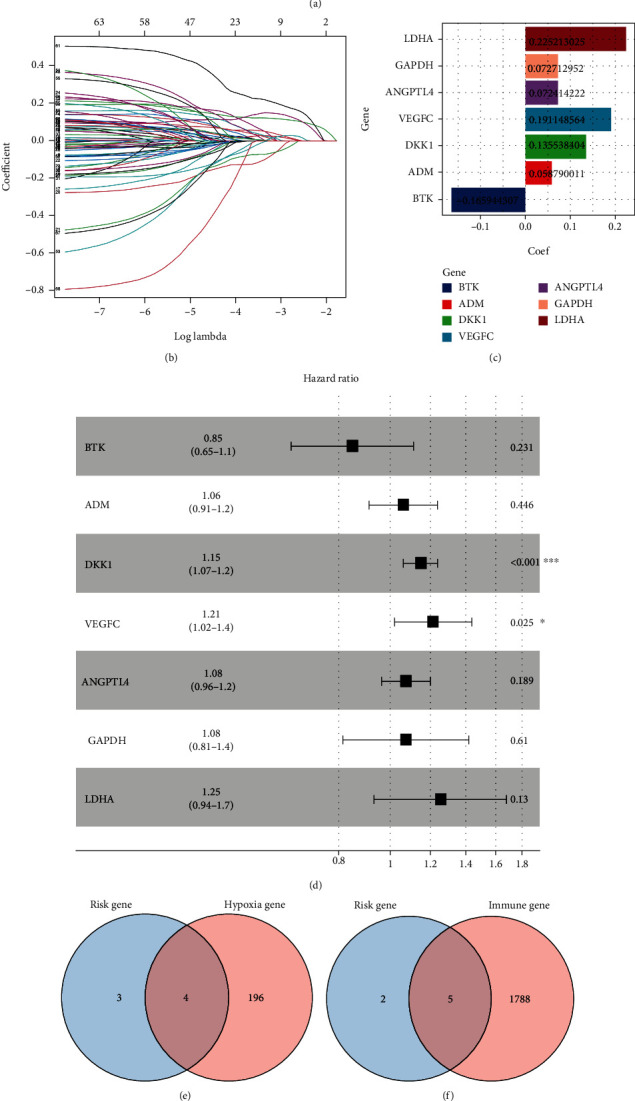
Calculating CIHI. (a, b) Parameter reduction by LASSO algorithm, the knee point determined by the effects of log penalty coefficient on the partial likelihood deviance was used to select the final parameters. (c, d) The coefficients of 7 final parameters obtained from Cox regression analysis. (e) Immune-related genes in 7 genes. (f) Hypoxia-related genes in 7 genes. (g) Hypoxia- and immune-related genes are common among 7 genes.

**Figure 3:**
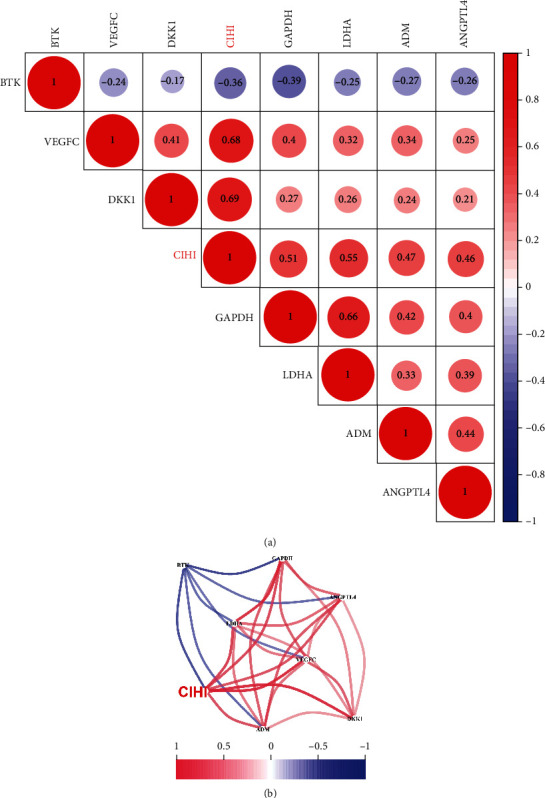
Correlation between CIHI and 7 genes. (a) Bubble plot of correlation between CIHI and 7 genes involved in the formula. (b) Network plot of correlation between CIHI and 7 genes involved in the formula. Red is positive, blue is negative.

Considering that hypoxia may influence the immune cell infiltration and the immune response, the combined analysis of hypoxia and immunity may have potential prognostic value and indicate the status of the tumor microenvironment (TME). Therefore, 68 prognostic genes were applied to the LASSO-Cox regression model to construct the CIHI formula in TCGA dataset. Seven genes were selected according to the LASSO regression analysis, and corresponding coefficients were generated at the optimum *λ* which is -2.81 (Figures 2(a) and 2(b)). In addition, the coefficient of each gene was further elaborated by multivariate Cox regression analysis to calculate CIHI (Figures 2(c) and 2(d)). CIHI = LDHA expression × 0.2252 + GAPDH expression × 0.0727 + ANGPTL4 expression × 0.0724 + VEGFC expression × 0.1911 + DKK1 expression × 0.1355 + ADM expression × 0.0588 + BTK expression × −0.1659. Among the 7 genes, ANGPTL4, GAPDH, LDHA, and ADM belong to hypoxia-related genes (Figure 2(e)), while immune-related genes are BTK, ADM, DKK1, VEGFC, and ANGPTL4 (Figure 2(f)). Interestingly, ANGPTL4 and ADM belong to both hypoxia-related genes and immune-related genes (Figure 2(g)). The CIHI of each patient was calculated according to this formula in TCGA, GSE68465, and GSE72094 cohorts. Subsequently, the three cohorts were divided into the high- and low-CIHI groups according to the median CIHI of TCGA cohort. In addition, Spearman correlation results showed that the CIHI was significantly correlated with the selected 7 genes, as shown in Figure 3(a). In addition, the correlation network showed that there was more red line than blue line, indicating that CIHI was positively correlated with most genes (Figure 3(b)). From the above analysis, we constructed a composite indicator and identified two subgroups of LUAD patients. Correlation analysis showed that CIHI was correlated with hypoxia-related genes, suggesting that CIHI might reflect hypoxia in TME.

These results suggested that hypoxia is associated with immune responses in the microenvironment. Therefore, this new scoring method, CIHI, may indicate the hypoxia immune status of patients.

### 3.3. High CIHI Represents Hypoxia^high^-Immunity^low^ in LUAD Patients

**Figure 4:**
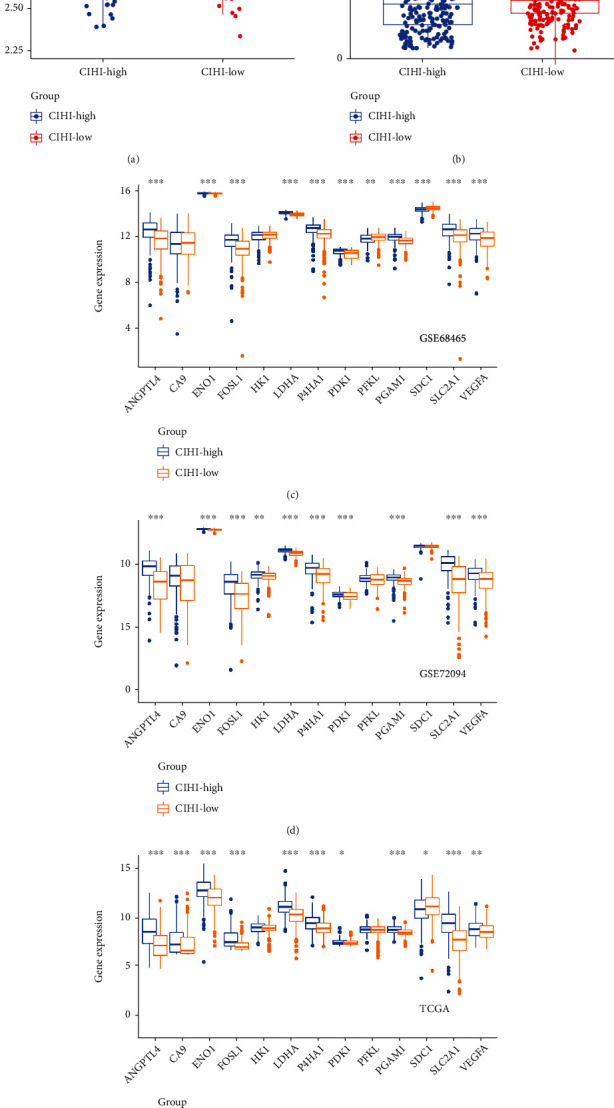
CIHI represents different immune and hypoxia states. PD-L1 expression in (a) GSE72094 and (b) TCGA cohorts in different CIHI score. Differential expression of hypoxia markers in (c) GSE68465, (d) GSE72094, and (e) TCGA cohorts. ^∗^*p* < 0.05, ^∗∗^*p* < 0.01, and ^∗∗∗^*p* < 0.001.

We further attempted to verify the association between CIHI and hypoxia. In a previous study, hub expression profiles associated with hypoxia in cancer were identified. We first compared key hypoxia-related characteristics in the high-risk and low-risk groups. Subsequent analyses described the 13-gene expression levels of the two phenotypes in three cohorts to reflect different hypoxia states. The expression of ANGPTL4, ENO1 FOSL1, LDHA, P4HA1, PDK1, PGAM1, SLC2A1, and VEGFA was significantly increased in the high-risk group of three cohorts. This may indicate that hypoxia-induced angiogenesis is more common in the high-risk group, suggesting that our patients with low CIHI have lower hypoxia levels (Figures 4(c)–4(e)). In addition to the GSE68465 cohort (without this gene annotation), we also examined PD-L1 expression in each cohort. Interestingly, patients with low CIHI had lower hypoxia but higher PD-L1 levels (Figures 4(a) and 4(b)). Therefore, we suggested that high CIHI represents hypoxia^high^-immunity^low^ in LUAD patients.

### 3.4. CIHI Levels and Immunoresponse Markers in LUAD

**Figure 5:**
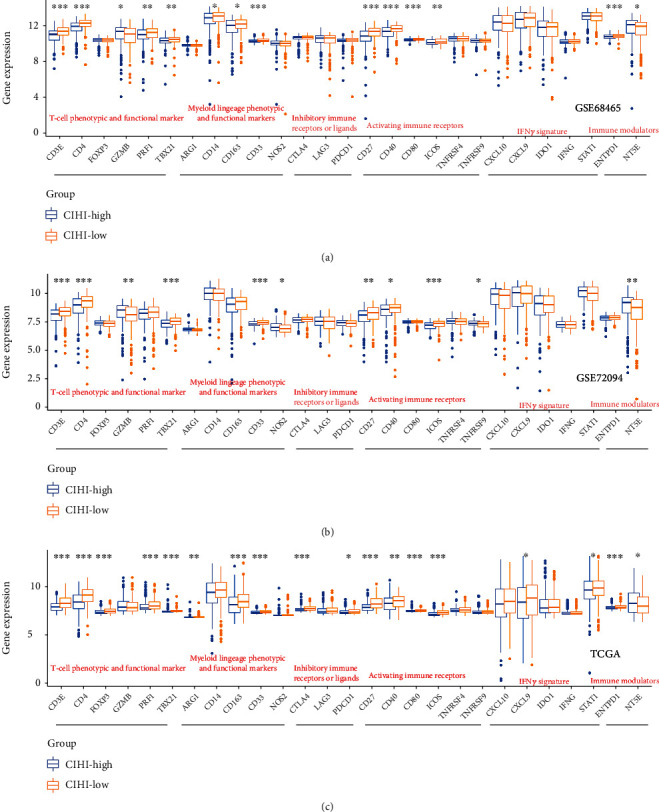
CIHI levels and immunoresponse markers in LUAD. T cell phenotypes and functional markers, myeloid lineage phenotypic and functional markers, inhibitory immune receptors or ligand markers, activating immune receptor markers, IFN*γ* signature markers, and immune modulator markers in (a) GSE68465, (b) GSE72094, and (c) TCGA cohorts based on CIHI score. ^∗^*p* < 0.05, ^∗∗^*p* < 0.01, and ^∗∗∗^*p* < 0.001.

To further promote CIHI function as an indicator of immune response, a set of immune response markers was also targeted to high-CIHI (hypoxia^high^-immunity^low^) and low-CIHI (hypoxia^low^-immunity^high^) phenotypes. Our results showed patterns of immune response between different phenotypes. In general, patients with hypoxia^high^-immunity^low^ exhibited a suppressive immune microenvironment compared with patients with hypoxia^low^-immunity^high^. This finding is consistent with the fact that these signals are responsible for regulating protumor or antitumor activity. In three cohorts, T cell phenotypes and functional markers, including CD3E, CD4, GZMB, and TBX21, were expressed at higher levels in the hypoxia^low^-immunity^high^ groups. Similarly, the differential genes in myeloid lineage phenotypic and functional markers, inhibitory immune receptors or ligand markers, activating immune receptor markers, IFN*γ* signature markers, and immune modulator markers were all highly expressed in the hypoxia^low^-immunity^high^ group (Figures 5(a)–5(c)). These results showed that there was a complex immune response in the low-CIHI group.

### 3.5. CIHI Levels Affect the Tumor Microenvironment

**Figure 6:**
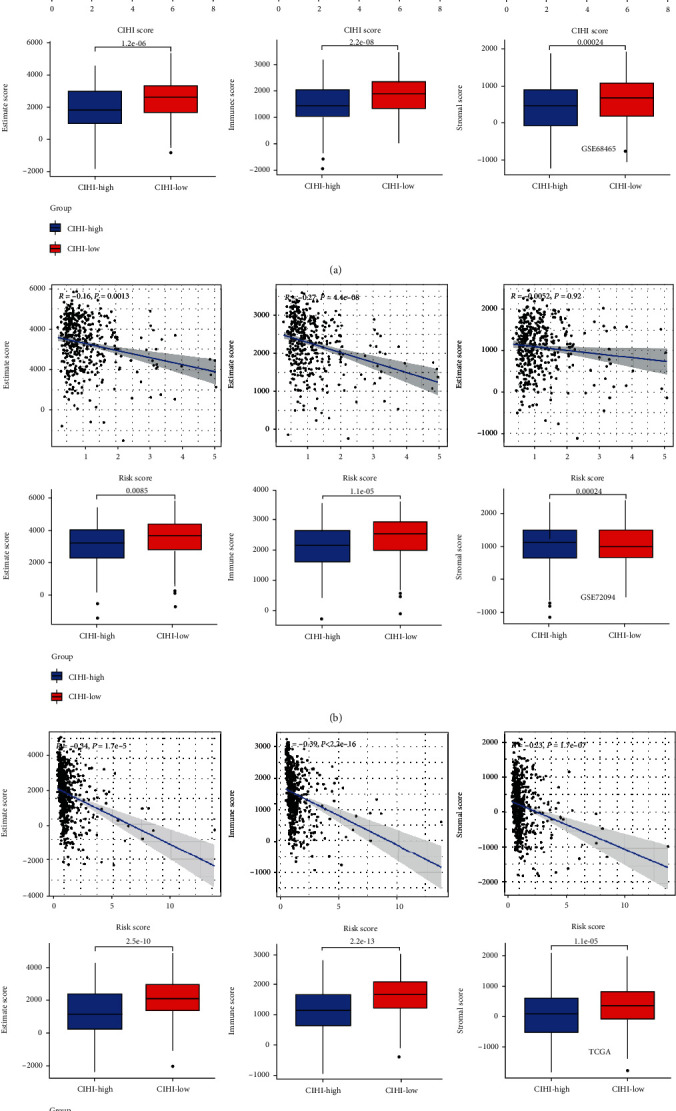
CIHI levels affect the tumor microenvironment. The stromal score was calculated between two CIHI groups using the ESTIMATE algorithm in (a) GSE68465, (b) GSE72094, and (c) TCGA cohorts. ^∗^*p* < 0.05, ^∗∗^*p* < 0.01, and ^∗∗∗^*p* < 0.001. ESTIMATE: Estimation of STromal and Immune cells in MAlignant Tumor tissues using Expression data.

Further results showed the characteristics of CIHI and its correlation with TME in each cohort. Interestingly, CIHI was negatively correlated with stromal score, immune score, and estimate score in three cohorts. It suggested that CIHI may also be associated with stromal non-immune-related components, such as fibroblasts (Figure 6). In addition, the overall landscape of TME was estimated using the ESTIMATE algorithm. All three scores were significantly higher in hypoxia^high^-immunity^low^ patients.

### 3.6. Benefits of CIHI for Clinical Application

**Figure 7:**
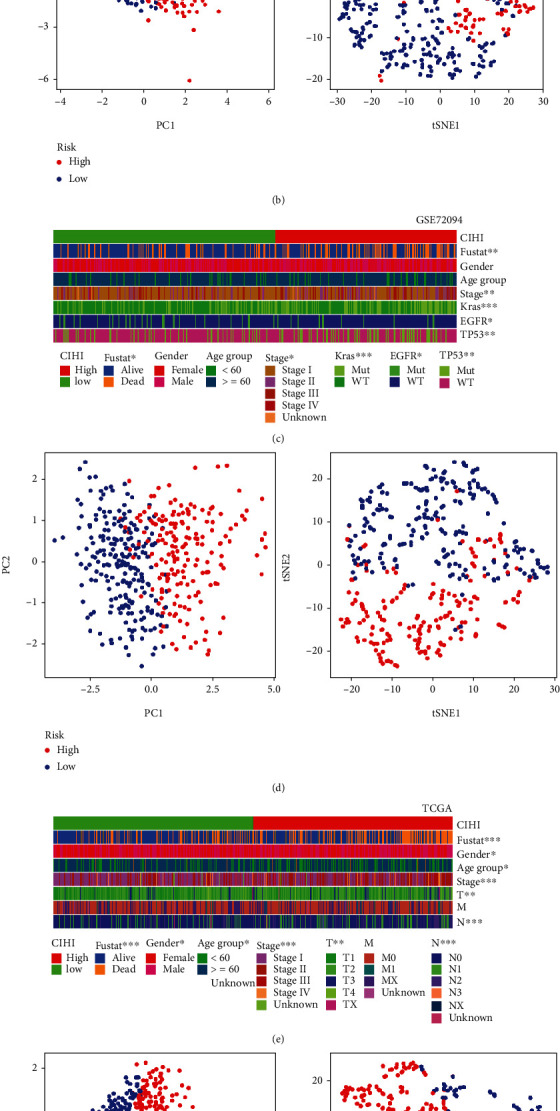
Composite heatmaps of CIHI and clinicopathological features in LUAD patients. (a) Heatmap of differences in clinicopathological factors and high- and low-CIHI groups in the GSE68465 cohort. (b) PCA analysis and t-SNE analysis in the GSE68465 cohort. (c) Heatmap of differences in clinicopathological factors and high- and low-CIHI groups in the GSE72094 cohort. (d) PCA analysis and t-SNE analysis in the GSE72094 cohort. (e) Heatmap of differences in clinicopathological factors and high- and low-CIHI groups in TCGA cohort. (f) PCA analysis and t-SNE analysis in TCGA cohort. ^∗^*p* < 0.05, ^∗∗^*p* < 0.01, and ^∗∗∗^*p* < 0.001.

**Figure 8:**
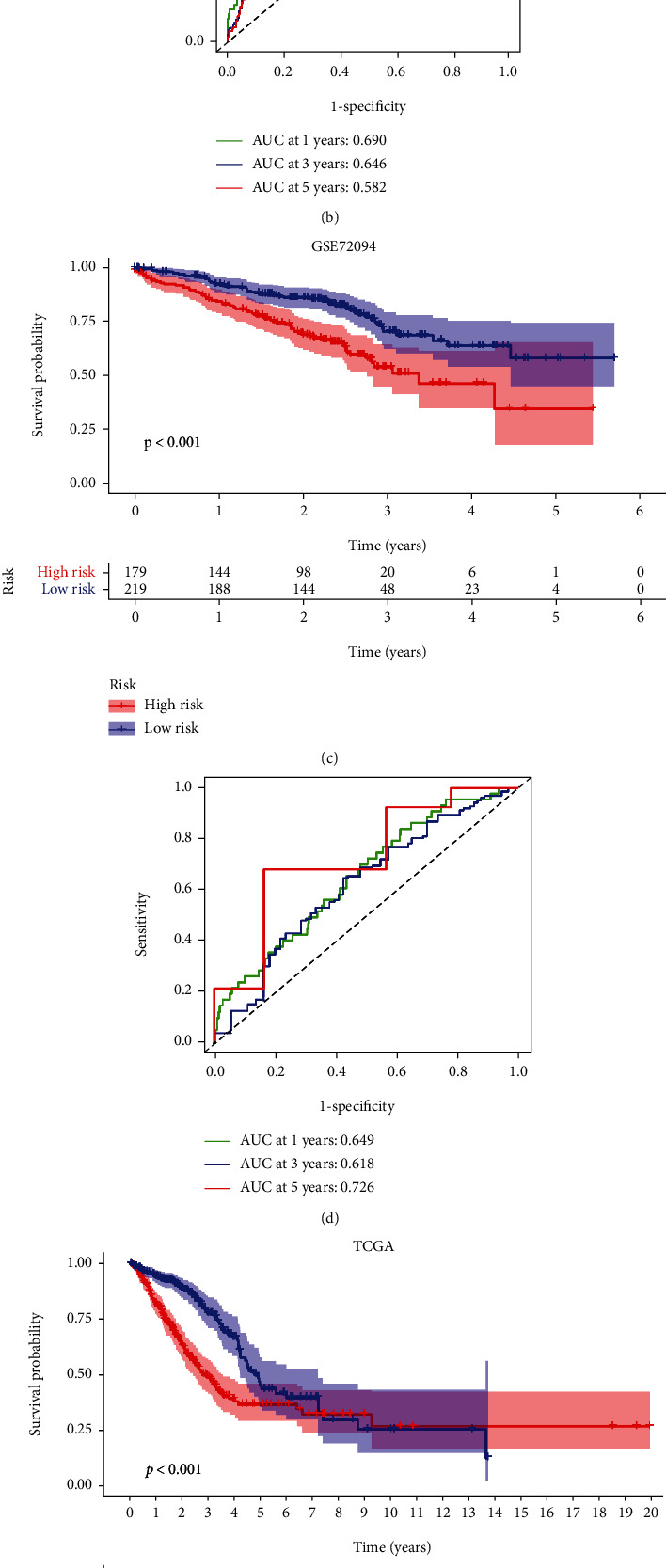
ROC and survival analysis in GEO and TCGA cohorts. Kaplan-Meier survival analysis of different CIHI scores in (a) GSE68465, (c) GSE72094, and (e) TCGA cohorts. ROC analysis based on CIHI in (b) GSE68465, (d) GSE72094, and (f) TCGA cohorts.

How to choose the appropriate tool or score for early diagnosis and treatment of cancer remains a key clinical issue. Previous studies have shown that hypoxia and changes in immune status are prominent features of malignant tumors. First of all, in PCA and t-SNE analysis, we found that three cohorts including TCGA (Figure 7(f)), GSE68465 (Figure 6(b)), and GSE72094 (Figure 6(d)) could be well distinguished and presented discrete distribution. In addition, we analyzed the correlation between CIHI score and various clinicopathological factors. In the GSE68465 cohort, the heatmap revealed that CIHI scores were significantly correlated with survival status, T stage, and grade (*p* < 0.05), as shown in Figure 7(a). Meanwhile, in the GSE72094 cohort, which contained information about the genetic mutation, the heatmap revealed that CIHI scores were significantly correlated with survival status, stage, KRAS, EGFR, and TP53 (*p* < 0.05), as shown in Figure 7(c). In addition, CIHI scores were associated with all clinical factors except M staging in TCGA cohort (*p* < 0.05), as shown in Figure 7(e). In addition, we further explored the predictive power of CIHI on survival status. Intriguingly, in three cohorts, a high CIHI score represented a poorer prognosis, with a significantly shorter survival time (*p* < 0.05), as shown in Figures 8(a), 8(c), and 8(e). In addition, we also conducted in-depth analysis of CIHI in predicting survival at different times. ROC analysis showed that CIHI had good predictive value for survival at 1, 3, and 5 years in different cohorts (AUC > 0.6), as shown in Figures 8(b), 8(d), and 8(f).

### 3.7. Validation of CIHI in the ICI Cohort

**Figure 9:**
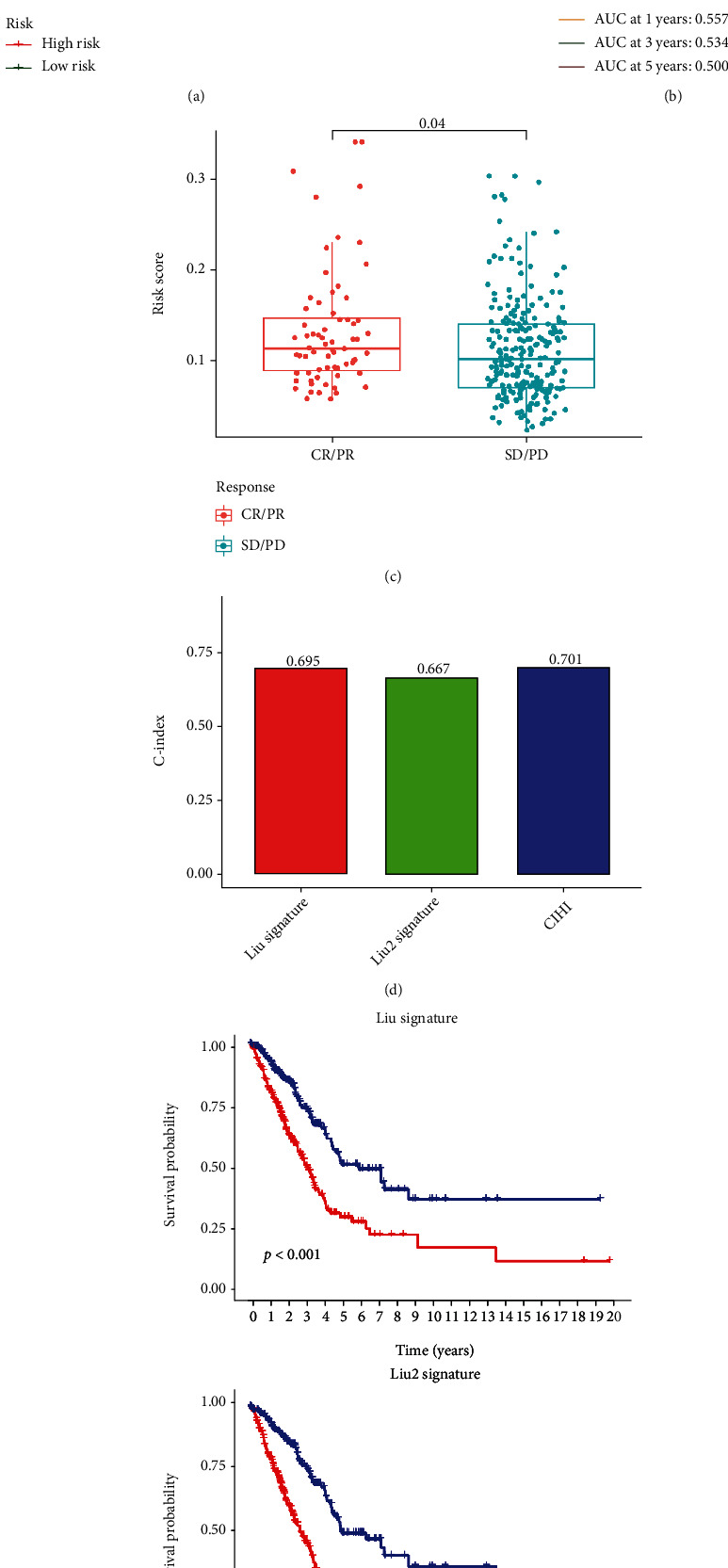
Validation of CIHI in ICI treatment cohort. (a) Kaplan-Meier survival analysis of anti-PD-L1 cohort. (b) ROC analysis of 1, 3, and 5 years in anti-PD-L1 cohort. (c) Difference of CIHI in different treatment response groups. (d) Comparison of *C*-index of different risk signatures. (e) Kaplan-Meier survival analysis about Liu and Liu2 signature.

We assessed the prognostic value of CIHI in the cohort treated with anti-PD-L1 (IMvigor). The results showed that low CIHI had better OS than low-CIHI patients (Figure 9(a)). Meanwhile, it can be found that in CR/PR and SD/PD cohorts, there are significant differences in CIHI (Figure 9(c)). Unfortunately, our CIHI may be a poor predictor of survival at 1, 3, and 5 years (Figure 9(b)), but the role of CIHIs in assessing the response to ICI treatment cannot be ignored. Finally, the prognostic value of CIHI was compared with other risk signatures [18, 19]. *C*-index results showed that CIHI had the strongest predictive performance (Figure 9(d)). In addition, it should not be ignored that other risk signatures can also stratify the risk of LUAD patients (Figure 9(e)).

## 4. Discussion

Although there have been advancements in treating advanced LUAD, such as immunotherapy, there are still many difficulties for researchers and clinicians to conquer. After all, this is an advanced stage of the disease and improving the OS of such patients remains difficult; studies have shown that the 5-year OS rate in patients with advanced LUAD is even lower than 20% [20]. Therefore, the current goal is to research and develop biomarkers as soon as possible to predict the prognosis of LUAD patients correctly, though in many ways understanding the correlation between tumor metabolism and immune cell infiltration inside TME is useful and development of therapeutics [21]. Although researchers have created many various approaches to define and quantify the immunological aspects of LUAD in contemporary studies, such as models and prognostic biomarkers, attention to the extracellular microenvironment, such as the effects of pH and hypoxia on cancer cells, remains modest [22]. In the study, we constructed a formula as follows: CIHI = LDHA expression × 0.2252 + GAPDH expression × 0.0727 + ANGPTL4 expression × 0.0724 + VEGFC expression × 0.1911 + DKK1 expression × 0.1355 + ADM expression × 0.0588 + BTK expression × −0.1659. Patients were divided into groups according to high and low CIHI, and expression profiles of hypoxia markers and immune markers were analyzed in different groups. CIHI was used to confirm that patients with high CIHI represented a state of hypoxia^high^-immunity^low^, which had worse overall survival. And most importantly, we also discussed the evaluation value in the immune microenvironment and clinical application value of CIHI.

The combined status of immune response and hypoxia in the TME is rarely noted. Some of the conclusions of this study are the same as those of previous studies; hypoxia promotes CD8+ T cell effects and migratory function, demonstrating that hypoxia has diverse functions in tumor cells and immune cells [23]. We also found more expression of immune cell markers in patients with hypoxia^high^. The significance of aberrant metabolism in tumor growth has gotten a lot of attention in recent years. To date, researchers have extensively explored and studied the expression patterns of metabolic enzymes in several cancer types, including prostate, breast, colorectal, gastric, and liver cancers [24]. We can continue to evaluate and investigate TME cell infiltration mediated by metabolic enzymes using a range of acceptable statistical methods in future studies thanks to the ongoing development of current sequencing technologies. In this study, CIHI was negatively correlated with stromal score, immune score, and estimate score in three cohorts. It suggested that CIHI may also be associated with stromal non-immune-related components, such as fibroblasts. In addition, the overall landscape of TME was estimated using the ESTIMATE algorithm. All three scores were significantly higher in hypoxia^high^-immunity^low^ patients. This indicates that the score has a robust potential to evaluate TME. Interestingly, cancer cells can emit various chemokines, which then attract monocytes to the tumor [25]. Within the tumor tissue, recruited monocytes quickly transform to become TAMs, inhibiting T cell activation and proliferation while also lowering their antigen expression ability [26]. TAMs have been proven in previous research to promote tumor proliferation and angiogenesis by enriching in hypoxic zones and secreting cytokines [27]. Therefore, our CIHI score can also reflect the level of immune evasion in LUAD tissues on the secondary side.

There are still some limitations of our study that are worth noting. The bioinformatics results, for starters, have been validated using TCGA and GEO samples. However, we were unable to conduct a second external validation, because we lacked the sufficient funding to sequence LUAD patients in our hospital. Second, we only used the ESTIMATE algorithm to corroborate our findings for the association between CIHI and TME, and we will need to conduct more experiments in the future to confirm our conclusion. In conclusion, this study developed and validated a hypoxia-immune formula that can guide hypoxia modifier treatment and immunotherapy in LUAD.

## Data Availability

The following information was supplied regarding data availability. Data is available at TCGA (https://portal.gdc.cancer.gov/) and GEO database (https://www.ncbi.nlm.nih.gov/geo/).
